# The exploration of anti-*Vibrio parahaemolyticus* substances from *Phellodendri Chinensis Cortex* as a preservative for shrimp storage

**DOI:** 10.3389/fmicb.2022.1004262

**Published:** 2022-09-13

**Authors:** Huifang Zheng, Yang Liu, Jing Cai, Miao Zhang, Ying Wen, Lei Guo

**Affiliations:** ^1^Jiangsu Key Laboratory of Marine Bioresources and Environment, Co-innovation Center of Jiangsu Marine Bio-industry Technology, Jiangsu Ocean University, Lianyungang, China; ^2^Jiangsu Key Laboratory of Marine Biotechnology, School of Food Science and Engineering, Jiangsu Ocean University, Lianyungang, China

**Keywords:** *Phellodendri Chinensis Cortex*, *Vibrio parahaemolyticus*, antibacterial activity, ultrasonic-assisted extraction, response surface methodology

## Abstract

This study aimed to optimize the ultrasonic-assisted extraction of the anti-*Vibrio parahaemolyticus* substances of *Phellodendri Chinensis Cortex* (ASPC), identify their active substances, and investigate their application in shrimp storage. The ultrasonic-assisted extraction conditions of ASPC were optimized through a single-factor experiment combined with response surface methodology. The optimal parameters were the ethanol concentration of 81%, the ultrasonic power of 500 W, the temperature of 80°C, the extraction time of 23 min, and the liquid/solid ratio 25 ml/g. The antibacterial zone diameter of the obtained extract determined by agar well diffusion method was 15.56 ± 0.22 mm, which was not significantly different from the predicted value (15.92 mm). Berberine was identified as one of the main chemical components of ASPC through high-performance liquid chromatography combined with standard control. The minimum inhibitory concentrations of ASPC and berberine determined by the tube dilution method were 0.25 and 0.03 mg/ml, respectively. The application of ASPC in shrimp storage showed that it could effectively inhibit the proliferation of *V. parahaemolyticus* on shrimps. This report offers good prospects for the use of *Phellodendri Chinensis Cortex* as a potential preservative against *V. parahaemolyticus* in aquatic products.

## Introduction

*Vibrio parahaemolyticus* is a halophilic Gram-negative opportunistic pathogen that is often found in coastal environments and marine-cultured fish, shrimps, and shellfish (Guo et al., 2022a). It may cause inflammation and congestion on the surface of cultured prawns and marine fish, as well as acute hepatopancreatic necrosis in prawns, thus causing great economic losses to the aquaculture industry (Ge et al., 2019; Guo et al., 2022b). Meanwhile, it is also an important seafood-borne pathogen that usually causes food poisoning and acute gastroenteritis (Yu et al., 2016). In recent years, *V. parahaemolyticus* infections have become one of the most widely distributed and common diseases, and one of the most important public health safety issues worldwide (Wu et al., 2014; Xu et al., 2014; Zhong et al., 2019). Nisin, a preservative that is extensively utilized in the food industry, is mainly used to control Gram-positive bacteria. However, it lacks inhibitory activity against Gram-negative bacteria, such as *V. parahaemolyticus* (Liu et al., 2017). Therefore, finding green, safe, efficient, and low-toxicity preservatives for aquatic products is extremely urgent.

*Phellodendri Chinensis Cortex* is a traditional and commonly used Chinese herbal medicine in China and some other Asian countries. It is the dry bark of the Rutaceae family member *Phellodendron chinense* Schneid. and is customarily called “Chuan Huang Bai.” *Phellodendri Chinensis Cortex* contains a variety of chemical components such as alkaloids, flavonoids, and terpenes, which have anti-cancer, antioxidant, antibacterial, hypoglycemic, and cardiovascular protection activities (Kim et al., 2017; Sun et al., 2019; Wang et al., 2021). During the preliminary screening of Chinese herbal medicines with activity against aquatic pathogenic bacteria, we found that *Phellodendri Chinensis Cortex* has good inhibitory activity against *V. parahaemolyticus*.

Ultrasound-assisted extraction is a new, green, and rapidly developing technology that is suitable for expanding the extraction scale of biologically active compounds and improving extraction efficiency. It has been successfully applied for the extraction of active ingredients from plants and animals (Guo et al., 2014; Wen et al., 2018; Chmelová et al., 2020). Response surface methodology (RSM) is an effective statistical method for optimizing multivariate problems and has been successfully employed for the modeling and optimization of biochemical processes (Guo et al., 2017). To the best of our knowledge, the ultrasound-assisted extraction of anti-*V*. *parahaemolyticus* active substances from *Phellodendri Chinensis Cortex* has not been reported. Thus, this study aims to optimize the ultrasound-assisted extraction of the anti-*V. parahaemolyticus* substances of *Phellodendri Chinensis Cortex* (ASPC), identify their main active components, and investigate their potential application in shrimp storage.

## Materials and methods

### Materials and reagents

*Phellodendri Chinensis Cortex* was purchased from a local Chinese pharmacy in Lianyungang, and was crushed by a pulverizer, and passed through a 40-mesh sieve for use. *V. parahaemolyticus* 1.1997 was purchased from China General Microbiological Culture Collection Center (CGMCC). Mueller-Hinton Broth (MHB) and Mueller-Hinton Agar (MHA) medium were purchased from Hangzhou Best Biotechnology Co., Ltd. Berberine was purchased from Hefei Bomei Biotechnology Co., Ltd. Methanol (chromatographic grade) and other chemical reagents (analytical grade) were purchased from Sinopharm Chemical Reagent Co., Ltd.

### Single factor experiment

One gram of *Phellodendri Chinensis Cortex* powder was weighed, placed in a 100 ml conical flask, and extracted with the SK8210LHC ultrasonic cleaner (40 kHz, Shanghai Kedao Ultrasonic Instrument Co., Ltd) under condensation reflux. The conical flask was fixed in the center of the extractor, and the heating water level exceeded the water level of the conical flask. First, the effects of different ethanol concentrations on ASPC extraction were investigated. A total of 20 ml of ethanol/water (V/V) was added at different concentrations into 100 ml conical flasks. Ultrasonic extraction was performed at 400 W and 60°C for 10 min. The extract was filtered through a Buchner funnel and concentrated under reduced pressure. The anti-*V*. *parahaemolyticus* activity of the extract was determined by using the agar diffusion assay after it was redissolved in 20 ml of 80% ethanol.

Second, the effects of ultrasonic power on the extraction of ASPC were determined. A total of 20 ml of 80% ethanol was added into 100 ml conical flasks. Ultrasonic extraction was conducted at 60°C for 10 min at 200, 300, 400, and 500 W. The extract was filtered through a Buchner funnel and made up to 20 ml with 80% ethanol for determination of the anti-*V*. *parahaemolyticus* activity.

Third, the effects of different extraction temperatures on ASPC extraction were evaluated. A total of 20 ml of 80% ethanol was added into 100 ml conical flasks. Ultrasonic extraction was carried out at 500 W for 10 min at 40°C, 50°C, 60°C, 70°C, and 80°C. The extract was filtered through a Buchner funnel and made up to 20 ml with 80% ethanol for determination of the anti-*V*. *parahaemolyticus* activity.

Fourth, the effects of different extraction times on ASPC extraction were investigated. A total of 20 ml of 80% ethanol was added into 100 ml conical flasks. Ultrasonic extraction was conducted at 80°C and 500 W for 5, 10, 20, 30, and 40 min. The extract was filtered through a Buchner funnel and made up to 20 ml with 80% ethanol for determination of the anti-*V*. *parahaemolyticus* activity.

Finally, the effects of different liquid/solid ratios on the extraction of ASPC were investigated. A total of 10, 20, 30, 40, and 50 ml of 80% ethanol were added into 100 ml conical flasks. Ultrasonic extraction was performed at 80°C and 500 W for 20 min. The extract was filtered through a Buchner funnel and concentrated under reduced pressure. The extract was redissolved in 20 ml of 80% ethanol. Then, its anti-*V*. *parahaemolyticus* activity was determined.

### Box–Behnken design

BBD combined with RSM was used to optimize ASPC extraction. BBD consisting of 17 experiments with three factors and three levels was carried out with ethanol concentration (*X*_1_), time (*X*_2_), and liquid/solid ratio (*X*_3_) as three independent variables (Table 1). The diameter of the inhibition zone of *Phellodendri Chinensis Cortex* extract was the response variable (*Y*, mm). The mode of the system was evaluated by using Design Expert 7.0.0 software and the following second-order polynomial equations:


$$Y=\beta_{0}+\overset{3}{\underset{i=1}{\sum }}\;\beta_{i}X_{i}+\overset{3}{\underset{i=1}{\sum }}\;\beta_{ii}X_{i}^{2}+\overset{2}{\underset{i=1}{\sum }}\overset{3}{\underset{j=i+1}{\sum }}\beta_{ij}X_{i}X_{j}$$

where *Y* is the predicted response value; *β*_0_, *β*_i_, *β*_ii_, and *β*_ij_ are the regression coefficients in intercept, linear and quadratic terms; and *X_i_* and *X_j_* are independent factors.

**Table 1:** Box–Behnken experimental design with the independent variables.

No	Variable levels			Response
	*X*_1_ (ethanol, %)	*X*_2_ (time, min)	*X*_3_ (liquid/solid ratio, mL/g)	*Y* (inhibition zone, mm)
1	−1 (70)	−1 (10)	0 (20)	11.90
2	+1 (90)	−1 (10)	0 (20)	13.60
3	−1 (70)	+1 (30)	0 (20)	13.36
4	+1 (90)	+1 (30)	0 (20)	15.04
5	−1 (70)	0 (20)	−1 (10)	11.68
6	+1 (90)	0 (20)	−1 (10)	14.04
7	−1 (70)	0 (20)	+1 (30)	14.49
8	+1 (90)	0 (20)	+1 (30)	13.67
9	0 (80)	−1 (10)	−1 (10)	12.27
10	0 (80)	+1 (30)	−1 (10)	13.03
11	0 (80)	−1 (10)	+1 (30)	14.72
12	0 (80)	+1 (30)	+1 (30)	15.50
13	0 (80)	0 (20)	0 (20)	15.58
14	0 (80)	0 (20)	0 (20)	15.21
15	0 (80)	0 (20)	0 (20)	14.96
16	0 (80)	0 (20)	0 (20)	16.54
17	0 (80)	0 (20)	0 (20)	15.55

### Determination of the anti-*Vibrio parahaemolyticus* activity of the extract

The antibacterial activity of the extract against *V. parahaemolyticus* was determined by using the agar well diffusion assay in accordance with the reference method (Guo et al., 2019). Twenty milliliter of MHB medium was poured into a Petri dish with a diameter of approximately 90 mm. Oxford cups (outer diameter of approximately 8.0 mm) were placed on the medium, which was precoated with 100 μl of *V. parahaemolyticus* suspension (1 × 10^6^ CFU/ml). Under these conditions, a clear bacterial circle can be formed. Then, 200 μl of the extract (50 mg/ml) was added to the cup. The Petri dishes were incubated at 37°C for 24 h. The inhibition zone (mm) was measured with a digital Vernier caliper, and the average of three parallel runs was recorded as the anti*-V. parahaemolyticus* activity of the extract.

### Identification of the main chemical component of ASPC

The main chemical component of ASPC was identified through high-performance liquid chromatography (HPLC) with berberine as the standard (Guo et al., 2020). A YMC-pack ODS (250 mm × 10 mm, 5 μm) chromatographic column was used. The detection wavelength was 345 nm, the flow rate was 1.5 ml/min, and the injection volume was 20 μl. The elution method was as follows: 0.1% phosphoric acid water/methanol mixed solvent gradient elution, 0–45 min, 10–70% methanol; 45–55 min, 70–100% methanol; and 55–65 min, 100% methanol.

### Determination of minimum inhibitory concentration (MIC) and minimum bactericidal concentration (MBC)

The MICs of ASPC and berberine were firstly determined by using the agar diffusion method in accordance with the reference method (Xu et al., 2021). ASPC and berberine solutions of different concentrations were prepared through the double dilution method, and the assay was carried out in accordance with the above-mentioned antibacterial activity determination procedure. The MIC was recorded as the minimum concentration that can produce the inhibition zone.

The MICs of ASPC and berberine were determined through the tube dilution method again in accordance with the reference literature (Guo et al., 2020). A total of 4.9 ml of MHB medium, 50 μl of *V. parahaemolyticus* suspensions (1 × 10^6^ CFU/ml), and 50 μl of 100 times the final concentration (1, 0.5, 0.25, 0.12, 0.06, 0.03 mg/ml) of ASPC and berberine solutions were added into 20.5 cm × 2.5 cm test tubes. A total of 50 μl methanol was used to replace the sample solution to prepare the negative control. After shaking and incubation at 37°C for 24 h, the minimum concentration of a sample with no visible bacterial growth to the naked eye was regarded as the MIC. A total of 100 μl of culture solution was pipetted from each test tube without the bacterial growth and spread evenly on a sterile MHA medium. The Petri dishes were incubated at 37°C for 24 h, and the MBC was recorded as the minimum concentration of the drug solution without colony formation.

### Application of ASPC in shrimp storage

The stock solution (10 mg/ml) of ASPC dissolved in 70% ethanol was diluted to the concentration of 1.0 mg/ml with sterile deionized water. Shrimps were sterilized at 121°C for 20 min, dried with sterile paper, and divided into three groups. Then, each group of shrimps was aseptically transferred into diluted ASPC (sample group), 7% ethanol (solvent control group), or sterile water (blank control group). The shrimps were soaked and shaken for 5 min. After soaking, the shrimps (5.0–7.0 g) were drained and transferred aseptically into a fresh-keeping bag. A total of 100 μl of *V. parahaemolyticus* suspensions (1 × 10^6^ CFU/ml) was inoculated on shrimps in each bag and kept at 4°C and room temperature (20°C–25°C) for 7 days. On days 1, 3, 5, and 7, the two samples were taken out and ground under aseptic conditions, and sterile water was added to prepare a 20 ml suspension. A total of 100 μl of the suspension was properly diluted and spread evenly on MHA medium, cultured at 37°C for 24 h, and used for the counting of colony-forming units (Wu et al., 2016).

### Statistical analysis

All experiments were conducted in triplicate, and the results were expressed as the mean ± standard deviation of three replicates. The results were subjected to one-way analysis of variance (ANOVA) followed by a least significant difference test. *p* < 0.05 was considered a significant difference, and *p* < 0.01 was considered an extremely significant difference.

## Results

### Single-factor experiment

In this study, the ultrasound-assisted extraction of ASPC was optimized. The key parameters that independently affected the extraction efficiency of ASPC, such as ethanol concentration, ultrasonic power, temperature, time, and liquid/solid ratio, were investigated before the optimization experiment.

**Figure 1:**
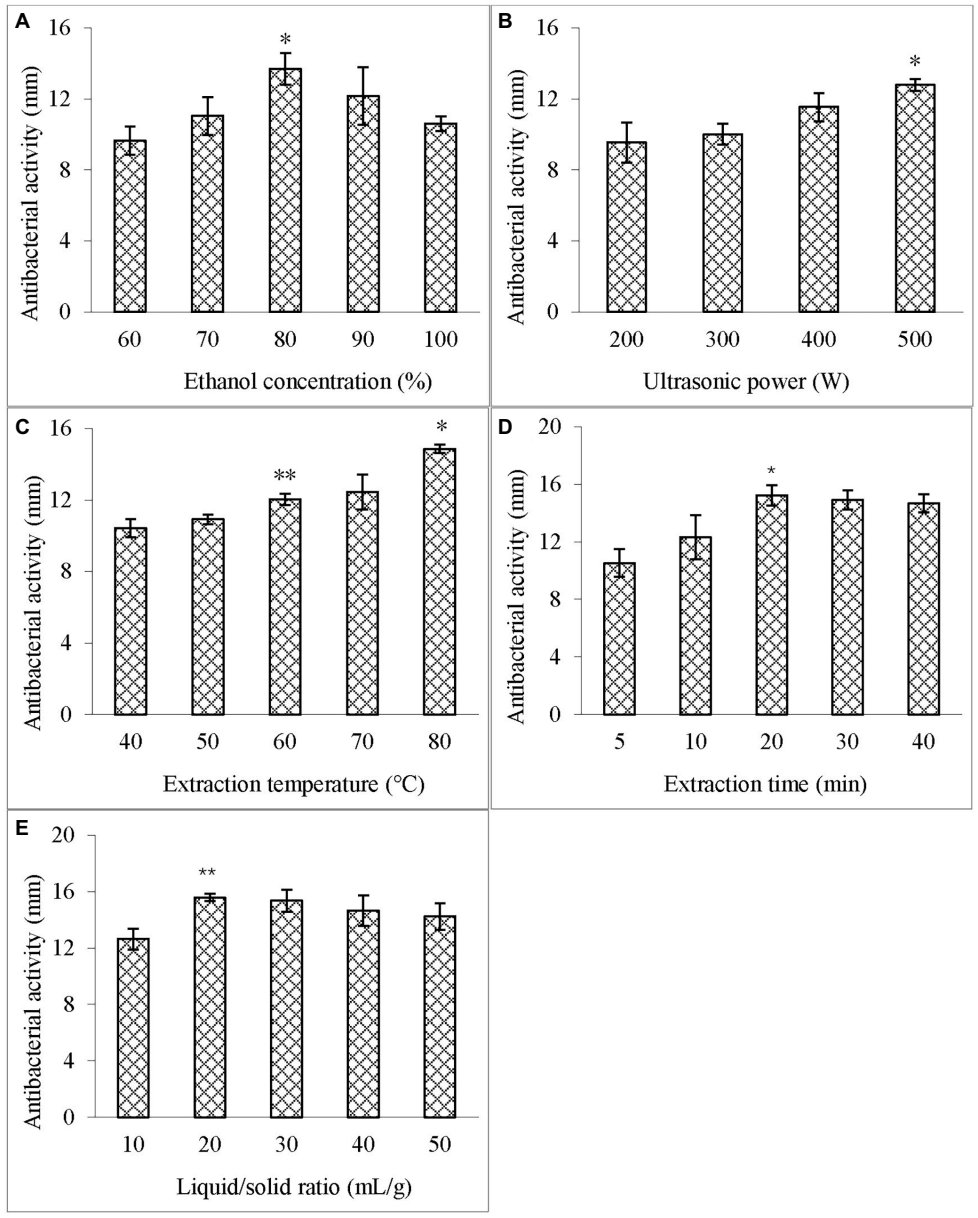
Effects of different single factors on the diameter of the inhibition zone of the extract. **(A)** Ethanol concentration; **(B)** Ultrasonic power; **(C)** Extraction temperature; **(D)** Extraction time; and **(E)** Liquid–solid ratio. Compared with the previous column, an asterisk (*) represents *p* < 0.05 and the double asterisk (**) represents *p* < 0.01.

The effects of different ethanol concentrations (60–100%, v/v) on the antibacterial activity of the extract against *V. parahaemolyticus* were examined at the liquid/solid ratio of 20:1 ml/g, 400 W, and 60°C with the extraction time of 10 min. Figure 1A illustrates that at the ethanol concentration of 80%, the diameter of the inhibition zone of the extract was the largest, indicating that ASPC has a certain hydrophobicity.

The effect of different ultrasonic powers (200–500 W) on the antibacterial activity of the extract against *V. parahaemolyticus* was studied at the ethanol concentration of 80%, liquid/solid ratio of 20:1 ml/g, and 60°C with the extraction time of 10 min. Figure 1B shows that as the ultrasonic power was increased, the diameter of the inhibition zone of the extract also increased, and reached the maximum value is at 500 W (100%). Therefore, the ultrasonic power was fixed at 500 W in the subsequent extraction.

The effect of different temperatures (40°C–80°C) on the antibacterial activity of the extract against *V. parahaemolyticus* was investigated at the ethanol concentration of 80%, liquid/solid ratio of 20:1 ml/g, and 500 W with the extraction time of 10 min. Figure 1C depicts that with the increase in the temperature, the diameter of the inhibition zone of the extract increased and was the largest at 80°C (100%). This result indicated that increasing the temperature within a certain range can increase the mass transfer coefficient and solubility of the active substances, thus contributing to the extraction of ASPC (Guo et al., 2016).

The effect of different extraction times (5–40 min) on the antibacterial activity of the extract against *V. parahaemolyticus* was analyzed at the ethanol concentration of 80%, liquid/solid ratio of 20:1 ml/g, 500 W, and 80°C. When the extraction time was increased from 5 min to 20 min, the diameter of the inhibition zone of the extract increased significantly, and the increase in the inhibition zone of the extract became no longer obvious with the prolongation of time (Figure 1D).

The effect of different liquid/solid ratios (10–50 ml/g) on the antibacterial activity of the extract against *V. parahaemolyticus* was studied at the ethanol concentration of 80%, 500 W, and 80°C with the extraction time of 20 min. Figure 1E indicates that the diameter of the inhibition zone increased significantly when the liquid/solid ratio was increased from 10 ml/g to 20 ml/g, but then stopped increasing as the ratio was further increased.

### Model fitting

On the basis of the single-factor experiment, ultrasonic power was fixed at 500 W and the extraction temperature of 80°C, the ethanol concentration of 80%, the extraction time of 20 min, and the liquid/solid ratio of 20 ml/g was used as the center points of three independent variables in the BBD for the optimization of the ultrasonic-assisted extraction process of ASPC. The coding and actual level of the three independent variables in the BBD and the response value (the diameter of the inhibition zone of the extract) are shown in Table 1. The following second-order polynomial equation that can explain the relationships between the test variables and the response variable was obtained through the multiple regression analysis of the experimental data:


$$\begin{array}{l} Y=15.57+0.62X_{1}+0.56X_{2}+0.92X_{3}-0.005X_{1}X_{2}\\ \;\;-0.80X_{1}X_{3}+0.005X_{2}X_{3}-1.25X_{1}^{2}-0.84X_{2}^{2}-0.85X_{3}^{2} \end{array}$$

The ANOVA is shown in Table 2, which illustrates that the *p* value of the established model is 0.0065, and the value of the lack-of-fit was 0.3859. These results indicated that the adaptability of the constructed model was very significant and can define the real behavior of the system. The coefficient of determination of the model was 0.9094, which showed that the model fully represented the true relationship between the independent variable and the response variable (Guo et al., 2014).

**Table 2:** Variance analysis of the effect of ethanol concentration, time and liquid/solid ratio on the anti-*V. parahaemolyticus* activity of the extract.

Source	Sum of squares	*df*	Mean square	*F* value	Prob > *F*	Significance
Model	28.79	9	3.20	7.81	0.0065	**
*X*_1_	3.03	1	3.03	7.39	0.0298	*
*X*_2_	2.46	1	2.46	6.02	0.0439	*
*X*_3_	6.77	1	6.77	16.54	0.0048	**
*X*_1_ *X*_2_	0.0001	1	0.0001	0.0002	0.9880	
*X*_1_ *X*_3_	2.53	1	2.53	6.17	0.0419	*
*X*_2_ *X*_3_	0.0001	1	0.0001	0.0002	0.9880	
*X*_1_^2^	6.59	1	6.59	16.11	0.0051	**
*X*_2_^2^	2.98	1	2.98	7.28	0.0307	*
*X*_3_^2^	3.02	1	3.02	7.37	0.0300	*
Lack of Fit	1.42	3	0.47	1.31	0.3859	

**Figure 2:**
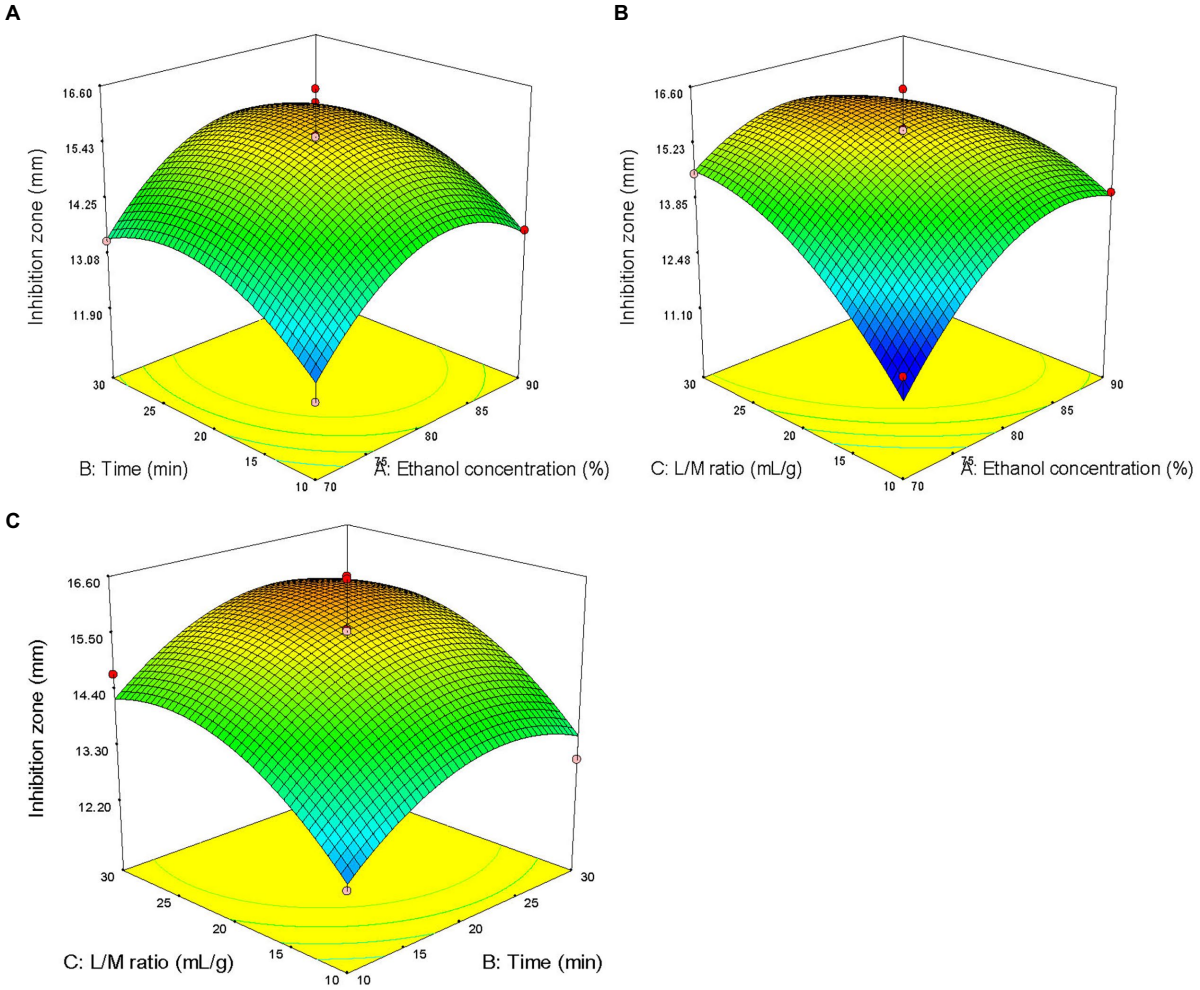
Response surface plots showing effects of pairwise factors on the antibacterial activity of the extract and their interaction. **(A)** Ethanol concentration and time (liquid/solid ratio was constant at 20: 1); **(B)** Ethanol concentration and liquid/solid ratio (the extraction time was constant at 20 min); and **(C)** time and liquid/solid ratio (ethanol concentration was constant at 80%).

The three factors investigated (ethanol concentration, extraction time, and liquid/solid ratio) all had significant effects on the antibacterial activity of the extract (*p* < 0.05). The primary and secondary order of affecting the antibacterial activity of the extract was liquid/solid ratio, ethanol concentration, and extraction time (Table 2). The three-dimensional response surface plot shown in Figure 2 is useful for viewing the interactive effects of factors on the response. The curved surface in Figure 2B is steep, indicating that the interaction between ethanol concentration and liquid/solid ratio is significant (*p* < 0.05), and the liquid/solid ratio has a greater impact on the antibacterial activity of the extract. Initially, with the increase of ethanol concentration, the antibacterial activity of the extract increased rapidly, and then began to decrease with the increase of ethanol concentration. Figures 2A,C show that the interactions between ethanol concentration and extraction time, extraction time and liquid/solid ratio are not significant. With the increase of ethanol concentration, the prolongation of extraction time, and the increase of liquid/solid ratio, the antibacterial activity of the extract increased first and then decreased.

The optimal process parameters predicted by the regression model were *X*_1_ = 81%, *X*_2_ = 23 min, and *X*_3_ = 25 ml/g, and the predicted maximum diameter of the inhibition zone was 15.92 mm. A verification experiment was carried out under the predicted optimal extraction conditions, namely, the ultrasonic power of 500 W, temperature of 80°C, ethanol concentration of 81%, liquid/solid ratio of 25 ml/g, and time of 23 min. The actual diameter of the inhibition zone of the extract was 15.56 ± 0.22 mm (*n* = 3), and did not significantly differ from the predicted value, indicating that the single-factor experiment combined with RSM for the optimization of ultrasonic-assisted extraction of ASPC is feasible.

### Identification of the main chemical component of ASPC

**Figure 3:**
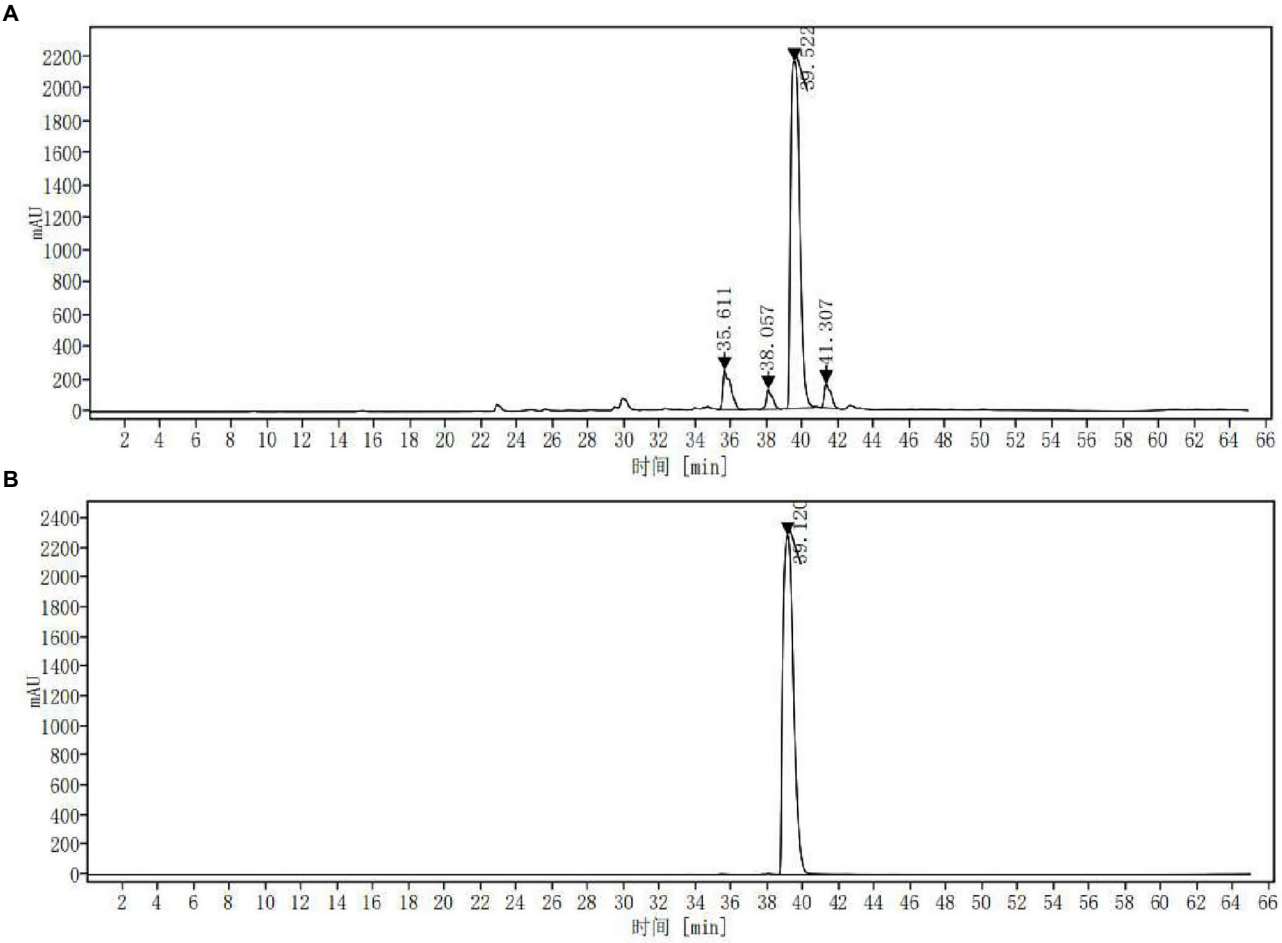
HPLC fingerprint chromatograms of ASPC **(A)** and berberine **(B)**.

The extract was concentrated and dried under reduced pressure to obtain ASPC. The yield of the solid ASPC was 11.18% ± 0.28% (*n* = 3). The HPLC fingerprint of ASPC showed that it contained four main chemical ingredients (Figure 3A) with the peak times of 35.611, 38.057, 39.522, and 41.307 min. In accordance with the absorption spectrum of the main chemical components and the existing literature (Chen et al., 2010), berberine was selected as the reference substance and a control experiment was carried out. Figure 3B shows that the retention time of berberine under the same conditions was 39.120 min. The substance in ASPC that peaked at 39.522 min was identified as berberine.

### Antibacterial activity of ASPC and berberine against *Vibrio parahaemolyticus*

The MICs and MBCs of ASPC and its main chemical component, berberine, against *V. parahaemolyticus* were determined through the agar diffusion and test tube methods. The MICs of ASPC and berberine against *V. parahaemolyticus* were 0.25 and 0.03 mg/ml, respectively, and the results determined by the two methods were consistent. The MBCs of ASPC and berberine for *V. parahaemolyticus* determined *via* the test tube method were 0.5 and 0.06 mg/ml, respectively (Table 3).

**Table 3:** Inhibitory activities of ASPC and berberine against *V. parahaemolyticus*.

Sample	MIC (mg/mL)		MBC (mg/mL)
	Agar diffusion method	Test tube method	Test tube method
ASPC	0.25	0.25	0.5
Berberine	0.03	0.03	0.06

### Application of ASPC in shrimp storage

**Figure 4:**
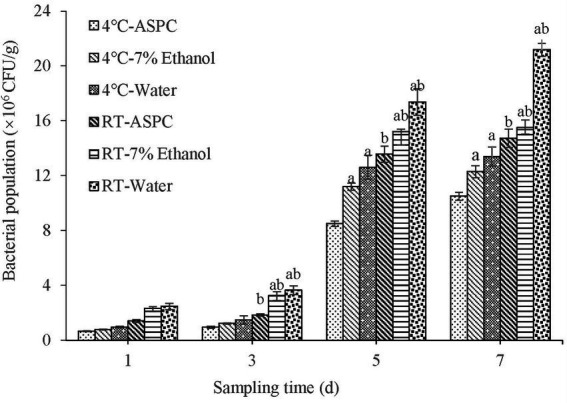
Colony numbers of raw shrimp treated with ASPC, 7% ethanol, and sterile water after 1, 3, 5, and 7 days at 4°C and room temperature. Compared with the ASPC group, the letter “a” represents *p* < 0.05. Compared with the 4°C group, the letter “b” represents *p* < 0.05.

The shrimps soaked in ASPC, 7% ethanol, and sterile water were inoculated with *V. parahaemolyticus* and stored at 4°C and room temperature (20°C–25°C). Samples were taken at regular intervals to quantify the colony-forming units in the shrimps. The results are shown in Figure 4. With the prolongation of the storage time, the number of microorganisms in each group gradually increased. Under the two conditions, the total number of colonies in the ASPC group was significantly lower than that in the 7% ethanol group and the sterile water group when the storage time was 3–7 days (*p* < 0.05). These results indicated that ASPC can effectively inhibit the growth of *V. parahaemolyticus* in shrimps. The number of colonies that formed on the shrimps at 4°C was significantly lower than that at room temperature (*p* < 0.05), indicating that the effect of ASPC is better at low temperatures than at high temperatures.

## Discussion

In China and other Asian countries, many plant-derived materials in traditional Chinese medicine have been used to fight infectious diseases for centuries. The preparation of antibacterial substances from traditional Chinese medicine as food preservatives against food-borne pathogens has the advantage of being environmentally friendly and relatively safe and has been extensively studied (Wang, 2014; Wu et al., 2016). *Phellodendri Chinensis Cortex* is a traditional Chinese medicine. Studies have shown that its decoction or alcohol extract has obvious antibacterial effects on *Staphylococcus aureus*, *Bacillus subtilis,* and *Streptococcus hemolyticus* (Li et al., 2019; Kim et al., 2020). However, whether *Phellodendri Chinensis Cortex* has inhibitory activity against *V. parahaemolyticus* has not been reported.

In this work, the ultrasonic-assisted extraction of ASPC was optimized through a single-factor experiment combined with RSM. Compared with the traditional maceration method, the ultrasonic-assisted extraction has the advantages of significantly shortening the extraction time and lowering the extraction temperature (Wen et al., 2018), which is confirmed by the results of this study (the optimized extraction temperature and time are 80°C and 23 min, respectively). Furthermore, berberine was identified as one of the main active molecules against *V. parahaemolyticus* in ASPC. It should be pointed out that the slight difference in retention time of ASPC and berberine under the same conditions should be attributed to the error caused by manual injection.

Berberine is an isoquinoline alkaloid that is mainly isolated and obtained from herbal plants, such as *Coptis chinensis*, *Berberis vulgaris*, *Hydrastis canadensis*, *Xanthorhiza simplicissima*, and *Phellodendron chinense* (Kim et al., 2017). Berberine is a natural bioactive molecule with multiple pharmacological activities. In addition to its anti-inflammatory, anti-tumor, anti-diabetic, anti-oxidant, hypolipidemic, and anti-osteoporosis effects, berberine can inhibit Gram-positive bacteria, such as *S. aureus*, *Streptococcus mutans*, *Streptococcus agalactiae*, *Streptococcus suis*, *Bacillus anthracis*, and *Enterococcus faecium* as well as Gram-negative bacteria such as *Actinobacillus pleuropneumoniae*, *Escherichia coli*, and *Shigella dysenteriae* (Wang et al., 2019; Zhang et al., 2021). This study confirmed that ASPC and its berberine have significant activities against the seafood-borne pathogen *V. parahaemolyticus* with the MICs of 0.25 and 0.03 mg/ml, respectively. The MIC value of berberine against *V. parahaemolyticus* is lower than that of the reported streptomycin sulfate (Guo et al., 2022b).

Previous studies have indicated that the extracts or compounds of herbal plants possess potent antibacterial properties against *V. parahaemolyticus*. For example, cinnamon extract (Lu et al., 2021), Chinese gall (*Galla chinensis*) extract (Wu et al., 2021), citral (Cao et al., 2021), and osmaronin (Guan et al., 2014) have MIC values of 6.25, < 0.2, 0.125 and 0.0207 mg/ml, respectively, for *V. parahaemolyticus*. These findings validate the potential application of *Phellodendri Chinensis Cortex* and its active compounds in the development of anti-*V*. *parahaemolyticus* agents.

Furthermore, many studies have reported that herbal plant extracts are used as preservatives for the storage of aquatic products. Wu et al. found that pomegranate peel (*Punica granatum* L) extract and Chinese gall extract can effectively inhibit the proliferation of *V. parahaemolyticus* and *Listeria monocytogenes* on cooked shrimp and raw tuna (Wu et al., 2016). Sae-Leaw and Benjakul soaked raw Pacific white shrimps in 1% cashew leaf extract for 30 min and stored them at 4°C for 12 days. They found that the total viable count and numbers of psychrophilic bacteria, *Pseudomonas*, H_2_S-producing bacteria, and *Enterobacter* on the treated shrimp were all lower than those on the control shrimp (*p* < 0.05) (Sae-Leaw and Benjakul, 2019). The present study found that ASPC can effectively inhibit the proliferation of microorganisms, such as *V. parahaemolyticus*, on shrimp at 4°C and room temperature. A further comparison of the effects of ASPC and commercial food preservatives on the changes in disease microorganisms and quality indicators during the storage of aquatic products will help determine whether ASPC can be used as a potential food preservative in the storage of aquatic products.

Although ASPC and its berberine exhibited potential biological effects against *V. parahaemolyticus*, the safety of their application should also be evaluated. Several studies have reported the safety of berberine in the clinical treatment of various diseases. Berberine had more adverse events than placebo in preventing the recurrence of colorectal adenomas, but no serious adverse events were observed (Fang et al., 2022). Berberine had good efficacy and safety in the treatment of type 2 DM, dyslipidemia, and hypertension (Lan et al., 2015; Zhao et al., 2020). These results indicate the good safety of ASPC and its berberine in the preservation of aquatic products.

In conclusion, the ultrasonic-assisted extraction of ASPC was optimized through a single-factor experiment and BBD combined with RSM. The optimal process parameters were the ultrasonic power of 500 W, the temperature of 80°C, the ethanol concentration of 81%, the liquid/solid ratio of 25 ml/g, and the extraction time of 23 min. Berberine was identified as one of the main active molecules of ASPC by using HPLC combined with standard comparison technology. The MICs of ASPC and berberine for *V. parahaemolyticus* were 0.25 and 0.03 mg/ml, respectively. ASPC can effectively inhibit the proliferation of *V. parahaemolyticus* on shrimp. This report offers good prospects for *Phellodendri Chinensis Cortex* as a potential preservative for *V*. *parahaemolyticus* in aquatic products.

## Data availability statement

The raw data supporting the conclusions of this article will be made available by the authors, without undue reservation.

## Author contributions

LG and HZ: designed the study and drafted the manuscript. HZ and YL carried out the experiments. JC and MZ: performed the data analysis, and the chart drawing. YW and LG: writing, review and editing. All authors have read and agreed to the published version of the manuscript.

## Funding

This work was financially supported by the Natural Science Foundation for Colleges and Universities in Jiangsu Province (19KJB350001) and Priority Academic Program Development of Jiangsu Higher Education Institutions (PAPD).

## Conflict of interest

The authors declare that the research was conducted in the absence of any commercial or financial relationships that could be construed as a potential conflict of interest.

## Publisher’s note

All claims expressed in this article are solely those of the authors and do not necessarily represent those of their affiliated organizations, or those of the publisher, the editors and the reviewers. Any product that may be evaluated in this article, or claim that may be made by its manufacturer, is not guaranteed or endorsed by the publisher.
